# Night contact lenses used for myopia – personal experience

**DOI:** 10.22336/rjo.2022.23

**Published:** 2022

**Authors:** Camelia Margareta Bogdănici, Irina Andreea Niagu, Alisa Bejan, Ștefan Tudor Bogdănici, Silvia Sălăvăstru

**Affiliations:** * Surgery II Department, Discipline of Ophthalmology, “Grigore T. Popa” University of Medicine and Pharmacy, Iași, Romania; ** “Stereopsis” Ophthalmological Clinic, Iași, Romania

**Keywords:** rigid gas permeable lenses, myopia reduction, quality of vision

## Abstract

**Objective:** The purpose of this study was to assess the quality of vision of patients who have chosen orthokeratology, and to identify different incidents that occur in patients who used this type of therapy.

**Materials and methods:** The study was conducted on a group of 10 patients who had a follow-up period of at least 4 years and presented regularly to ophthalmological check-ups. The clinical parameters that were analyzed are the following: ocular refraction before and after orthokeratology therapy, the appearance of corneal topography, the slit-lamp examination of the anterior segment of the eye, incidents determined by night contact lenses, as well as ocular comfort.

**Results:** Issues produced by night lenses occurred in two cases due to deficient hygiene and care and in one case due to disruption of lens wear. Menicon lenses were used in 7 cases and Precilens lenses were used in 3 cases. The initial visual acuity without correction was between 0.02 and 0.7, and after orthokeratology 8 out of 10 patients had a visual acuity of 1.

**Conclusions:** In our study, orthokeratology therapy demonstrated its efficiency in slowing myopia progression and no severe complication was observed during the follow-up period.

## Introduction

A contact lens (CL) represents a device placed over the cornea or sclera to correct ametropia, or it can be used for ophthalmological treatment purposes. An adequate polymeric material is needed to produce contact lenses. A polymer represents a large macromolecule formed of hundreds or even thousands of repeating molecules that are called monomers. The materials used for contact lens manufacturing are usually composed of polymer-hydrogel or silicone-hydrogel. CL were initially created from glass until the introduction of polymethylmethacrylate in the 1930s [**1**].

After it was discovered that polymethylmethacrylate caused corneal hypoxia, newer materials were created. The first gas permeable lens with cellulose acetate was created in 1937, cellulose acetate butyrate was introduced in 1974 and fluorosilicone acrylate material was developed in 1987. Gas permeable materials are typically produced from fluorosilicone acrylates. Gas permeable contact lenses are used for keratoconus treatment, for correcting high astigmatism, and are also used in orthokeratology [**1**].

The term orthokeratology was introduced in the 1950s, and overnight wear was approved in 2002 [**2**]. Orthokeratology demonstrated its efficiency in myopia and/ or myopic astigmatism reduction and is recommended especially in children and teenagers [**3**]. Moreover, orthokeratology decreases the elongation of the antero-posterior axis of the eyeball [**4**].

Orthokeratology still represents an expensive technique that can be dauting for some patients who will look for alternative means of treatment [**5**]. Prevention of complications requires adequate choice of patients and type of lenses [**6**].

In the case of night contact lenses use, corneal topography is mandatory before and during the treatment to follow-up on the efficiency of applanation [**7**]. Orthokeratology lens represent a gas permeable contact lens that improves temporary daytime visual acuity, after overnight use and wake up removal [**8**].

Orthokeratology lenses are composed of fluorosilicone acrylate materials and the Dk is between 100 and 163 [**9**-**11**].

Corneal topography is used in orthokeratology in order to analyze the corneal shape before treatment. Keratron Piccolo, used for corneal topography, can achieve axial and tangential topography [**12**]. Zeiss Atlas 9000 is another commonly used corneal topographer that can analyze different corneal parameters.

## Materials and methods

This research was reviewed by an independent ethical review board and is in conformity with the principles and applicable guidelines for the protection of human subjects in biomedical research. The informed consent was read and signed by all the patients or their parents and the study was conducted according to a based protocol. The study was approved by the Ethics Committee of Grigore T. Popa University of Medicine and Pharmacy, Iași, on August, 17, 2015. 

This study was a prospective one and it was performed on a group of 10 patients. Orthokeratology treatment was recommended for every patient. The study was performed in the “Stereopsis” Ophthalmological Clinic, Iași, with a follow-up interval of 6 years. 

The purpose of this study was to assess the quality of vision of patients who have chosen night contact lenses, and to identify different incidents that occur in patients who used this type of contact lenses.

The following clinical parameters were monitored for all patients: ocular refraction before and after orthokeratology therapy, the appearance of corneal topography, the slit-lamp examination of the anterior segment of the eye, incidents determined by night contact lenses, as well as ocular comfort. For visual acuity, we used Snellen chart and for refraction, we used the value of the spherical equivalent.

The criteria used for patient inclusion were the following: age over 7 years old, low, or moderate myopia < -8D, myopic astigmatism with cylinder value < 2.50D, absence of systemic or ocular allergies, acute inflammation of the anterior segment of the eye, dry eye disease, systemic underlying pathologies, follow-up for more than one year.

The criteria used for patient exclusion were the following: patients who could not adapt with night contact lenses, patients who no longer came for follow-up or came for follow-up less than one year.

Zeiss Atlas 9000 and Keratron Piccollo were used for corneal topography. Both offer the possibility of objective interpretation of ocular surface changes. 

## Results

The patients included in this study were between 9 and 42 years old, 70% of them being females. Our findings showed that 7 eyes had low myopia, 9 eyes - medium myopia and 4 eyes - high myopia. The initial visual acuity without correction was between 0.02 and 0.7, and after orthokeratology 8 out of 10 patients had a visual acuity of 1. Measurements were performed after at least 1 year treatment with night contact lenses. The VA after orthokeratology was measured in binocular vision and was similar with the one obtained with eye glasses.

The mean visual acuity in the right eye without correction before orthokeratology was 0.274 and after treatment was 0.97. For the left eye, the mean visual acuity without correction before orthokeratology was 0.234 and after treatment was 0.97. There was a statistical significance between the values of the visual acuity in right eye and left eye after orthokeratology (***P*** < 0.05). The mean values of objective refraction in spherical equivalent before treatment was -3.3 for the right eye and -3 for the left eye. After at least one year of night contact lens treatment, the mean value of objective refraction in spherical equivalent was -1.1 for the right eye and -1.475 for the left eye. There was a statistical significance between the values of refraction in both eyes after treatment with night contact lenses (***P*** < 0,05). Descriptive data of patients, refraction, and visual acuity before and after treatment are presented in **Table 1**-**3**.

**Table 1:** Descriptive data of patients and right eye refraction and visual acuity before treatment

No.	Gender	Age	Objective refraction RE	Refraction RE EqS	Initial VA RE
1	F	9	-4.25 sph -0.25 cyl/ 164	- 4.37	0.2
2	M	15	-0.75 sph -0.25 cyl/ 178	- 0.88	0.7
3	M	14	-4 sph -0.5 cyl/ 116	- 4.25	0.2
4	F	29	-1.25 sph -1.5 cyl/ 146	- 2	0.5
5	M	32	-2.75 sph -0.5 cyl/ 171	- 3	0.4
6	F	21	-6.5 sph -0.75 cyl/ 160	- 6.87	0.02
7	F	9	-4.5 sph – 1 cyl/ 164	- 5	0.2
8	F	42	-2.5 sph -0.25 cyl/ 115	- 2.62	0.4
9	F	15	-7 sph -0.25 cyl/ 170	- 7.12	0.02
10	F	28	- 5.5 sph -0.25 cyl /160	- 5.62	0.1
RE = right eye; EqS = spherical equivalent; VA = visual acuity;					

**Table 2:** Descriptive data of patients and left eye refraction and visual acuity before treatment

No.	Gender	Age	Objective refraction RE	Refraction RE EqS	Initial VA RE
1	F	9	- 5.25 sph -0.5 cyl/ 176	- 5.5	0.1
2	M	15	- 1.75 sph -0.25 cyl/ 4	- 1.87	0.5
3	M	14	- 4 sph -0.25 cyl/ 70	- 4.12	0.2
4	F	29	- 1.5 sph -1.25 cyl/ 62	- 2.12	0.5
5	M	32	- 2.75 sph -0.5 cyl/ 1	- 3	0.4
6	F	21	- 6 sph -0.75 cyl/ 170	- 6.37	0.02
7	F	9	- 5.5 sph -0.75 cyl/ 4	- 5.87	0.2
8	F	42	- 3.5 sph -0.25 cyl/ 171	- 3.62	0.3
9	F	15	- 8 sph -0.25 cyl/ 65	- 8.12	0.02
10	F	28	- 5.75 sph -0.25 cyl/ 2	- 5.87	0.1
LE = left eye; EqS = spherical equivalent; VA = visual acuity;					

**Table 3:** Visual acuity and spherical equivalent refraction after orthokeratology

No.	VA RE after orthokeratology	VA LE after orthokeratology	Refraction RE Eqs after orthokeratology	Refraction LE Eqs after orthokeratology
1	0.9	0.9	-0.5	-1.75
2	1	1	-0.25	-0.5
3	1	1	-1.25	-1.25
4	1	1	-0.5	-0.75
5	1	1	-0.25	-0.5
6	1	1	-1	-1.25
7	1	1	-0.75	-1.25
8	1	1	-0.75	-0.5
9	0.8	0.8	-2.25	-2.5
10	1	1	-1	-1
RE = right eye; LE = left eye; EqS = spherical equivalent; VA = visual acuity;					

We used Menicon and Precilens contact lenses in this study. Menicon lenses were used in 7 cases and Precilens lenses were used in 3 cases. The anterior segment examination revealed no significant modifications for all patients before and after treatment with orthokeratology.

**Fig. 1:**
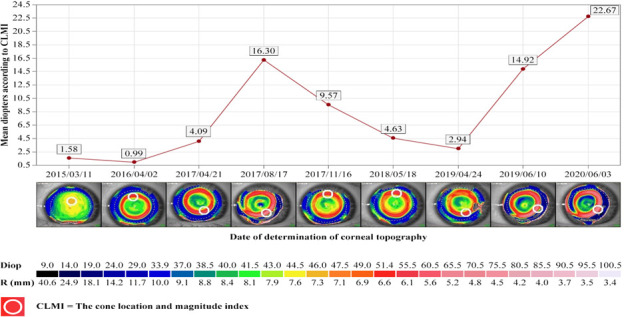
The evolution of CLMI in right eye (personal case report)

**Fig. 2:**
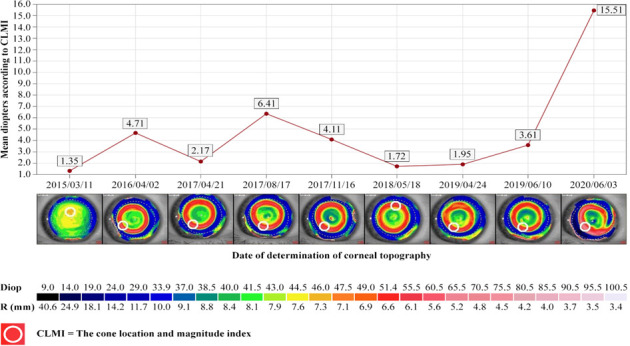
The evolution of CLMI in left eye (personal case report)

We evaluated the Cone Location and Magnitude Index, which was calculated on the available axial and tangential curvature. Cone Location and Magnitude Index represents an important index that can identify the presence or absence of a keratoconus pattern in corneal topography. The white circle on the red ring from the corneal topography represents Cone Location and Magnitude Index. The values in the graphics represent the average of the diopters according to Cone Location and Magnitude Index (**Fig. 1**, **Fig. 2**).

 In one case, topography of the cornea was performed with difficulty. Issues produced by night contact lenses wear occurred in 2 cases due to poor hygiene and care and in one case due to discontinuation of lens wear.

## Discussion

Myopia prevalence in early age is increasing all over the world and it represents an important public health issue. Apart from orthokeratology, there is evidence that atropine therapy is efficient in reducing myopia progression in children. The mechanism of atropine action is not completely understood, but it is believed that it is acting through a different path than orthokeratology. The association between orthokeratology and atropine could have an important benefic effect in reducing myopia progression [**13**]. In this study, we did not use this combined therapy, but only orthokeratology treatment.

Orthokeratology is used nowadays as a safe approach for myopia correction up to 6.00 D. More importantly, orthokeratology is an effective treatment for slowing myopia progression in children [**14**].

The contact lenses used for the patients in this study were Menicon and Precilens. Menicon lenses are created from a material called Siloxanylstyrene fluoromethacrylate (tisifilcon A) [**15**], while Precilens lenses are a Double Reservoir Lenses that are made of a Boston XO2 (hexafocon B) material [**9**].

Three out of the ten patients received Precilens lenses. One of them, a 15-year-old female, with spherical equivalent refraction in right eye -7.12 and in left eye -8.12, received this type of lens as initial therapy. In a paper by Zhou et al., it was concluded that orthokeratology can be beneficial in controlling or delaying high myopia progression [**16**].

Another female patient with a constant spherical equivalent refraction in the right eye -5.62 and in the left eye -5.87 for approximately 10 years, also received Precilens. 

The third patient was a 42-year-old female who performed refractive surgery on both eyes, but due to myopia recurrence she received Precilens. A 2020 paper conducted on adults with myopia, showed that orthokeratology represents a reliable therapy option for myopia reduction in adults [**17**].

A two-year Hong Kong paper performed on 35 children who used orthokeratology and a control group with the same number of patients who used single-vision spectacle lenses, concluded that the mean increase in axial length in the orthokeratology group was signiﬁcantly lower than in the control group [**18**].

A study conducted by Lee and col. on a 12-year-period showed that orthokeratology was efficient in slowing myopia progression [**19**].

In our study, the follow-up period was up to 6 years. Our results showed that orthokeratology had a significant role in slowing myopia progression in children, as well as in adults, which was similar with the previous studies.

In a 2016 paper, no major ocular side effects after orthokeratology therapy were reported. Only one patient presented difficulties adjusting the lens in the first months, but most of the patients followed all the hygiene indications. Moreover, all the included participants became more self-confident with the aid of the night contact lenses [**12**]. All these results were comparable with our data.

A study conducted by Chan using Menicon Z-Night contact lenses showed that toric periphery orthokeratology could be useful for high corneal astigmatism [**20**]. Another study conducted by Paune on 32 patients using toric orthokeratology Precilens showed that toric orthokeratology reduces refractive astigmatism [**21**].

One of the most common complications of night contact lenses wear is represented by corneal staining [**22**]. This complication was not observed in our study.

In a paper including 3800 eyes, 8.8% of patients presented visual acuity issues secondary to interrupted wear and 4% of them wore the contact lenses only for a brief period [**23**]. These findings were comparable with the ones from our study (patients who were excluded from our study).

Although preliminary results from different studies showed that axial elongation is slower for combined therapy with orthokeratology and 0.01% atropine, more research must be done to certify this aspect [**24**]. This combined therapy was not used in our study.

Another study showed that night contact lenses have a beneficial effect on accommodative function during the treatment and there was a significant association between improved accommodative accuracy and decreased axial elongation during the first six months of therapy [**25**].

A study conducted by Zhang and col. demonstrated the efficiency of orthokeratology in controlling progression of anisometropic myopia, because of the inhibition of the axial length elongation of monocular myopic eyes [**26**].

The major strength of this paper is represented by the results that showed that orthokeratology therapy had a major role in slowing the myopic progression in children, as well as in adults. This study was limited by the absence of evaluation of the anteroposterior axis and by the small study group. 

## Conclusion

In conclusion, in our study, orthokeratology therapy demonstrated its efficiency in slowing myopia progression and no severe complication was observed during the follow-up period. Our study demonstrated that the patients’ quality of vision improved significantly following orthokeratology, and 8 out of 10 patients had a visual acuity of 1 after using night contact lenses.

**Conflict of Interest Statement**

The authors state no conflict of interest. 

**Informed Consent and Human and Animal Rights statement**

Informed consent has been obtained from all the patients and the parents of the patients included in the study.

**Authorization for the use of human subjects**

Ethical approval: The research related to human use complies with all the relevant national regulations, institutional policies, it is in accordance with the tenets of the Helsinki Declaration and has been approved by the Ethics Committee of Grigore T. Popa University of Medicine and Pharmacy, Iași, Romania. 

**Acknowledgements**

None. 

**Sources of Funding**

None. 

**Disclosures**

None. 
